# Editorial: New therapeutic approaches for SARS-CoV-2/COVID-19

**DOI:** 10.3389/fimmu.2023.1276279

**Published:** 2023-08-24

**Authors:** Alfonso J. Rodriguez-Morales, Alexandre Naime Barbosa, Sergio Cimerman

**Affiliations:** ^1^ Clinical Epidemiology and Biostatistics, Faculty of Health Sciences, Universidad Científica del Sur, Lima, Peru; ^2^ Gilbert and Rose-Marie Chagoury School of Medicine, Lebanese American University, Beirut, Lebanon; ^3^ Infectious Diseases Department, Botucatu School of Medicine, UNESP, Botucatu, Brazil; ^4^ Institute of Infectious Diseases Emilio Ribas, São Paulo, Brazil

**Keywords:** SARS-CoV-2, COVID-19, emerging, coronavirus, emerging infectious diseases, global health, therapy, pandemic

After three years and a half since the pandemic due to the Severe Acute Respiratory Syndrome coronavirus 2 (SARS-CoV-2) causing Coronavirus Disease 2019 (COVID-19) started (1), there has been an enormous impact in terms of morbidity and mortality due to this emerging pathogen (2). Up to August 9, 2023, there have been more than 769.37 million cases, with 6.95 million deaths, as reported to the World Health Organization (WHO) (**Figure 1**). Multiple advances during this time have been vital in controlling and ceasing the pandemic condition and the recent declaration to lift the international public health emergency in May 2023. One of them is undoubtedly related to the impact of preventing multiple outcomes (including deaths) of efficacious, safe and effective globally deployed vaccines against SARS-CoV-2 (3, 4). As of August 9, 2023, 13,492 million vaccine doses have been administered (**Figure 1**).

**Figure 1:**
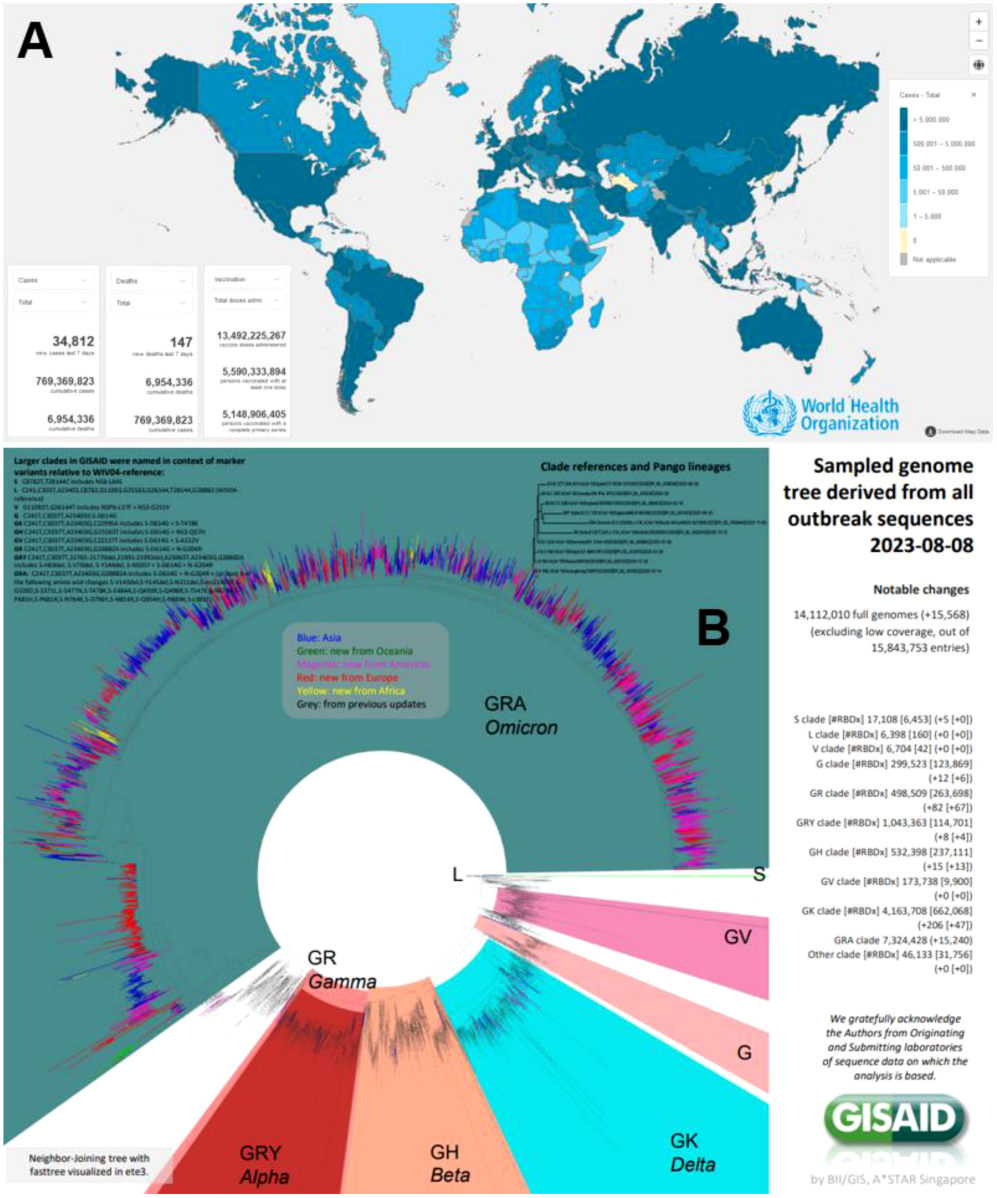
**(A)** COVID-19 global situation of cases, according to the WHO (https://covid19.who.int/). **(B)** Genomic distribution clades, lineages and variants of SARS-CoV-2 during 2020-2023 by GISAID (https://gisaid.org/).

Although that cases of COVID-19 still occur, there is still a considerable risk to vulnerable populations presenting comorbidities, immunosuppression, and elderly, among other conditions prone to severe disease and even death, especially in low and middle-income countries (5–7). In addition, although for primary vaccination schemes, there was high vaccination coverage, especially in low and middle-income countries, subsequent boosters were less popular. Consequently, coverages for them (3^rd^, 4^th^ and 5^th^ doses) (8) were significantly lower compared to primary schemes (9). Even in Sub-Saharan Africa, such figures are worse. But also, in Latin America and Asia, there are multiple countries where 5^th^ dose boosters are not yet available, despite the importance of the Omicron subvariants or lineages (**Figure 1**). Then, cases will continue and need medical management, including hospitalisation and sometimes admitting patients to the intensive care units for multiple reasons, requiring pharmaceutical evidence-based treatment (10, 11).

For those reasons, new efficacious, safe and effective therapeutical approaches for SARS-CoV-2/COVID-19 are still needed. In the current Research Topic (RT) about it, multiple studies and reviews unveiled different strategies for the management of infection and its associated complications. This RT includes 16 articles developed by authors from various countries (China, Germany, India, Italy, Malaysia, Poland, Spain, and the USA). Such articles covered original articles performing basic (Zekri et al.) and clinical studies (Ticinesi et al.), including animal models (Tian et al.), bioinformatics, observational and experimental designs (Wang Z. et al.), as well as systematic and scoping reviews, also with meta-analysis, during this Omicron times (Xu et al.). In addition to traditional therapeutic approaches, some of them discuss potential alternative therapies for COVID-19 (Rizvi et al.), primarily used in Asia (Wang Z. et al.), and also looking new potential pharmacological targets (Huang et al.). Focused on therapeutic potential, also some of the studies assess pathogenesis (Gao et al.) and physiopathological mechanisms (Lu et al.) related to different COVID-19 complications and therapeutic approaches (Liu et al. ), performing comparisons with acute respiratory distress syndrome (ARDS) (Llanos et al.) and sepsis (Li et al.). Some studies focused on the usefulness of monoclonal antibodies (Liew et al.), including also new site-specific targeted drug delivery (Zielińska et al.). Immunotherapies, such as convalescent plasma, are also included in this RT (Qian et al.).

The systematic review of Qian et al. from China on convalescent plasma for COVID-19 patients concluded that more double-blinded randomised clinical trials are needed to investigate the efficiency of convalescent plasma among patients in the initial stage of COVID-19, especially those who were within three days from symptoms onset and without detectable neutralising antibodies at enrolment.

Another systematic review on the topic, from Liew et al. from Malaysia and the USA concluded that the preclinical evidence suggests that bebtelovimab, a monoclonal antibody, would be a potential treatment for COVID-19 amidst viral evolution. Bebtelovimab has comparable efficacy to other COVID-19 therapies without evident safety concerns.

Another monoclonal antibody, tocilizumab, has been coated with solid lipid nanoparticles loaded with cannabidiol as a novel drug delivery strategy for treating COVID-19 and assessed in a review from Zielińska et al. from Poland, Bulgaria, Austria and Portugal.

Other immunomodulators, such as type I interferons (IFNs), inhibit the replication of both DNA and RNA viruses at different stages of their replication cycles and effect activating immune cell populations to clear infections; type I IFNs are directly antiviral agents and have been proposed for therapy in COVID-19. An n open prospective cohort study from Xu et al. from China showed that IFN α-2b spray shortened the viral shedding time of the Omicron SARS-CoV-2 variant when administrated within three days since the first positive test for SARS-CoV-2.

Further interventions, such as the gamma-aminobutyric acid (GABA)-receptor agonists, are promising, as an animal model in SARS-CoV-2-infected mice reduces pneumonitis severity, viral load, and death rate (Tian et al.), as shown by Tian et al. from USA.

At the molecular level, other articles assessing the expression and function of circular RNAs during severe acute COVID-19 showed their importance in the regulation of the inflammatory response, viral replication, immune evasion, and cytokines induced by SARS-CoV-2 infection (Gao et al.). An original study described the engineering of an optimised angiotensin-converting enzyme 2 (ACE2) fusion protein, designated ACE2-M, which comprises a human IgG1 Fc domain with abrogated Fc-receptor binding linked to a catalytically-inactive ACE2 extracellular domain that displays increased apparent affinity to the B.1 spike protein. The affinity and neutralisation capacity of ACE2-M is unaffected or even enhanced by mutations in the spike protein of viral variants. In contrast, a recombinant neutralising reference antibody and antibodies present in the sera of vaccinated individuals lose activity against such variants. With its potential to resist viral immune escape, ACE2-M appears to be particularly valuable in the context of pandemic preparedness towards newly emerging coronaviruses (Zekri et al.).

A review focused on oral GS-441524 derivatives (VV116, ATV006, and GS-621763; version 2.0, targeting highly conserved viral RdRp) that would be considered as game-changers in treating COVID-19 because oral administration has the potential to maximise clinical benefits, including decreased duration of COVID-19 and reduced post-acute sequelae of SARS-CoV-2 infection, as well as limited side effects such as hepatic accumulation (Wang Z. et al.). That review summarises the current research related to the oral derivatives of GS-441524 and provides essential insights into the potential factors underlying the controversial observations regarding the clinical efficacy of remdesivir; overall, it offers an effective launching pad for developing an oral version of GS-441524.

In physiopathology, original research investigated the role of cellular stress and binding-immunoglobulin protein (BiP) (Llanos et al.) in the modulation of the ARDS inflammatory response in samples from COVID-19 patients and a mouse model of ARDS. The authors demonstrate that BiP levels correlate with the severity of ARDS. Furthermore, they showed that the localisation of BiP on the cell surface is increased in the immune cell lineages during ARDS proportionally to the severity of the inflammatory response and identify a network of proteins that mediate this pathological process. Such results support using BiP as a prognosis biomarker of severe pneumonia and offer a new therapeutic strategy for diseases with ARDS, such as COVID-19.

ARDS is still a matter of concern in COVID-19 patients. Consequently, a bioinformatics study focused on understanding the mutual differentially expressed genes (DEGs) for the patients with COVID-19, ARDS and sepsis for functional enrichment, pathway analysis, and candidate drugs analysis. Such candidate drugs in the study may contribute to effectively treating COVID-19 (Li et al.). Another similar bioinformatic study also reports similar findings. Based on enrichment analysis of common DEGs, many pathways closely related to inflammatory response were observed, such as the Cytokine-cytokine receptor interaction pathway and NF-kappa B signalling pathway. In addition, protein-protein interaction networks and gene regulatory networks of common DEGs were constructed, and the analysis results showed that the Integrin Subunit Alpha M (ITGAM) may be a potential key biomarker base on regulatory analysis. Furthermore, a disease diagnostic model and risk prediction nomogram for COVID-19 were constructed using machine learning methods. Finally, potential therapeutic agents, including progesterone and emetine, were screened through drug-protein interaction networks and molecular docking simulations (Lu et al.). Today, Computers are key in assessing potential therapies, including alternative medicine effects and traditional Chinese approaches. In another study, the authors used various network pharmacology methods combined with CADD techniques to reveal the diversity of potential targets and therapeutic pathways for QFPD against COVID-19. They found that RBP4, IL1RN, TTR, FYN, SFTPD, TP53, SRPK1, and AKT1 are highly related to COVID-19. QFPD could act on multiple pathways, including viral process, immunodeficiency, RNA polymerase, Sphingolipid signalling pathway, and taste transduction. The results showed that QFPD has “multi-component, multi-target, and multi-pathway” characteristics in regulating inflammation, viral infection, cellular damage, and immune responses (Wang Z. et al.).

Traditional but natural medicine also may provide potential therapeutic approaches. Because of this, an original study assessed the pharmacological potential of *Withania somnifera* (L.) Dunal (WS) and *Tinospora cordifolia* (Willd.) Miers on the experimental models of COVID-19, T cell differentiation, and neutrophil functions (Rizvi et al.). The results indicate that WS promoted the immunosuppressive environment in the hamster and hACE2 transgenic mice models and limited the worsening of the disease by reducing inflammation, suggesting that WS might be useful against other acute viral infections. That study thus provided preclinical efficacy data to demonstrate a robust protective effect of WS against COVID-19 through its broader immunomodulatory activity.

A molecular docking study (Huang et al.) showed that vitamin D possessed effective binding activity in COVID-19. Overall, the authors showed vitamin D’s possible molecular mechanisms and pharmacological targets for treating COVID-19.

Finally, clinical studies are also included in the RT. One of them focused on comparing the characteristics and outcomes of patients admitted with confirmed COVID-19 in the same season during the first (March 2020) and the third pandemic wave (March 2021, dominance of SARS-CoV-2 B.1.1.7 lineage) in an internal medicine ward of a large teaching hospital in Italy (Ticinesi et al.). Despite the higher virulence of B.1.1.7 lineage, authors detected milder clinical presentation and improved mortality in patients hospitalised during the third COVID-19 wave, with the involvement of younger subjects. The reasons for this discrepancy are unclear but could involve the population effect of vaccination campaigns conducted primarily in older frail subjects during the third wave. The second study (Liu et al.) constructed a prone ventilation management scheme for patients with severe coronavirus disease 2019 (COVID-19). It analysed its application effect, finding that its application can standardise and promote the implementation of prone ventilation, improve the quality of care, and improve the patient prognosis of COVID-19 patients.

## Author contributions

AR-M: Conceptualization, Data curation, Formal Analysis, Investigation, Methodology, Writing – original draft, Writing – review & editing. AB: Writing – original draft, Writing – review & editing. SC: Writing – original draft, Writing – review & editing.

## References

[1] Zhu N Zhang D Wang W Li X Yang B Song J. A novel coronavirus from patients with pneumonia in China, 2019. New Engl J Med (2020) 382(8):727–33. doi: 10.1056/NEJMoa2001017 PMC7092803 31978945

[2] Rodriguez-Morales AJ Cardona-Ospina JA Gutierrez-Ocampo E Villamizar-Pena R Holguin-Rivera Y Escalera-Antezana JP. Clinical, laboratory and imaging features of COVID-19: A systematic review and meta-analysis. Travel Med Infect Dis (2020) 34:101623. doi: 10.1016/j.tmaid.2020.101623 32179124 PMC7102608

[3] Solante R Alvarez-Moreno C Burhan E Chariyalertsak S Chiu NC Chuenkitmongkol S. Further implications on the global real-world vaccine effectiveness against SARS-CoV-2. Expert Rev Vaccines (2022) 21(9):1355–7. doi: 10.1080/14760584.2022.2110073 35968671

[4] Solante R Alvarez-Moreno C Burhan E Chariyalertsak S Chiu NC Chuenkitmongkol S. Expert review of global real-world data on COVID-19 vaccine booster effectiveness and safety during the omicron-dominant phase of the pandemic. Expert Rev Vaccines (2023) 22(1):1–16. doi: 10.1080/14760584.2023.2143347 36330971

[5] Cimerman S Chebabo A Cunha CAD Rodriguez-Morales AJ. Deep impact of COVID-19 in the healthcare of Latin America: the case of Brazil. Braz J Infect Dis (2020) 24(2):93–5. doi: 10.1016/j.bjid.2020.04.005 PMC7177128 32335078

[6] Cimerman S Chebabo A Cunha CAD Rodriguez-Morales AJ. One year after the arrival of COVID-19 in Latin America: what have we learned in Brazil and other countries? Braz J Infect Dis (2021) 25(2):101571. doi: 10.1016/j.bjid.2021.101571 33741322 PMC9392129

[7] Rodriguez-Morales AJ Gallego V Escalera-Antezana JP Mendez CA Zambrano LI Franco-Paredes C. COVID-19 in Latin America: The implications of the first confirmed case in Brazil. Travel Med Infect Dis (2020) 35:101613. doi: 10.1016/j.tmaid.2020.101613 32126292 PMC7129040

[8] Owusu-Dampare F Bouchnita A. Equitable bivalent booster allocation strategies against emerging SARS-CoV-2 variants in US cities with large Hispanic communities: The case of El Paso County, Texas. Infect Dis Model (2023) 8(3):912–9. doi: 10.1016/j.idm.2023.07.009 PMC10400804 37547263

[9] Urrunaga-Pastor D Bendezu-Quispe G Herrera-Añazco P Uyen-Cateriano A Toro-Huamanchumo CJ Rodriguez-Morales AJ. Cross-sectional analysis of COVID-19 vaccine intention, perceptions and hesitancy across Latin America and the Caribbean. Travel Med Infect Dis (2021) 41:102059. doi: 10.1016/j.tmaid.2021.102059 33848692 PMC8063600

[10] Barbosa AN Chebabo A Starling C Pérez C Cunha CA de Luna D. Pan-American Guidelines for the treatment of SARS-CoV-2/COVID-19: a joint evidence-based guideline of the Brazilian Society of Infectious Diseases (SBI) and the Pan-American Association of Infectious Diseases (API). Ann Clin Microbiol Antimicrob (2023) 22(1):67. doi: 10.1186/s12941-023-00623-w 37550690 PMC10408214

[11] Saaavedra-Trujillo CH Gutiérrez AB Rodríguez-Morales AJ Narváez Mejía ÁJ García Peña ÁA Giraldo Montoya ÁM. Consenso Colombiano de atención, diagnóstico y manejo de la infección por SARS-COV-2/COVID-19 en establecimientos de atención de la salud - Recomendaciones basadas en consenso de expertos e informadas en la evidencia. Infectio (2020) 24(S3):1–102. doi: 10.22354/in.v24i3.851

